# Supplementary material for: Impact of sequencing depth and read length on single cell RNA sequencing data of T cells
Source: Sci Rep. 2017 Oct 6;7:12781. doi: 10.1038/s41598-017-12989-x (PMC5630586; doi:10.1038/s41598-017-12989-x)

Supplementary Notes

Impact of sequencing depth and read length on single cell RNA sequencing data of T cells.

Authors: Simone Rizzetto[1,2], Auda A. Eltahla[1,2], Peijie Lin[3,4], Rowena Bull[1,2], Andrew R. Lloyd[1,2], Joshua W. K. Ho[3,4], Vanessa Venturi[5], Fabio Luciani[1,2,*]

Affiliation:

1 School of Medical Sciences, UNSW Australia

2 Viral Immunology Systems Program, Kirby Institute for Infection and Immunity, UNSW Australia

3 Victor Chang Cardiac Research Institute, Sydney, NSW, Australia.

4 St. Vincent's Clinical School, UNSW Australia

5 Infection Analytics Program, Kirby Institute for Infection and Immunity, UNSW Australia

- To whom correspondence should be addressed. Email: Luciani@unsw.edu.au

Validation of TCR reconstruction using TraCeR

In order to validate the TCR reconstruction from publically available scRNA-seq dataset, a second available software program, TraCeR, was utilized.

TraCer algorithm is as outlined in the following steps: i) scRNA-seq reads are aligned with bowtie against a priori list of TCR sequences generated from the IMGT database (a combinatorial recombinome that encompass the repertoire of VDJ genes known to date); ii) aligned reads to those TCR sequences are assembled using Trinity [1]; iii) the resulting alignments are then interrogated via Igblast to extract TCR sequences; iv) a filtering step is performed whereby only the top two expressed TCR sequences are retained.

The output of TraCeR was taken prior to the filtering step (step iv above), as to maintain the same definition of successful TCR reconstruction utilized for the VDJPuzzle (see Material and Methods in the main document). Therefore the TCR sequences obtained from TraCeR were inputted into MigMap and tested against the IMGT database. As for VDJPuzzle, we considered a successful TCR reconstruction if at least one TCR sequence (α or β) was identified from the data, in frame, complete, and without stop codons.

Regarding the methodological differences between VDJPuzzle and TraCeR, an important difference is the fact that contrary to TraCeR, VDJPuzzle does not require a priori reference list of TCR, i.e. a combinatorial recombinome that encompass the repertoire of VDJ genes known to date via the IMGT database. Instead VDJPuzzle performs *de novo* assembly with all the reads that align (one of the pair) to the constant region of the TCR. Tracer on the other hand performs *de novo* assembly only with the reads that have successfully aligned to the combinatorial recombinome that has been set a priori.

The results from TraCeR were consistent with the VDJpuzzle result (Table S1). Table S2 shows the confusion matrices for TCRα and TCRβ reconstruction between the two methods. The minor dissimilarities observed between the two approaches are likely due to differences in the algorithms implemented in the two programs.

Table S1. The success rate of reconstructing full-length T-cell receptors (TCR) using TraCeR.

Dataset	Number of cells	Average reads length (nt)	Average number of PE reads ($x10^6$ reads)	TCRα success rate (%)TraCeR	TCRβ success rate (%)TraCeR	TCRαβ success rate (%)TraCeR
1	54	145	8.4	81.48	85.19	81.48
2	12	215	3.5	100.00	100.00	100.00
3	399	125	2.5	92.98	93.48	91.23
4	269	100	3.7	89.59	94.42	86.62
5	100	25	1.5	0.00	0.00	0.00
6	272	100	4.3	80.88	91.54	80.88
7	106	32	1.2	2.83	0.00	0.00
8	93	75	1.6	90.32	92.47	86.02

Table S2. Confusion matrices for both TCRα and TCRβ reconstructions between VDJPuzzle and TraCeR.

TCRα		VDJPuzzle	
		Successful	Unsuccessful
TraCeR	Successful	902 (69%)	62 (4.7%)
	Unsuccessful	131 (10%)	210(16%)

TCRβ		VDJPuzzle	
		Successful	Unsuccessful
TraCeR	Successful	957 (73%)	58 (4%)
	Unsuccessful	94 (7%)	196 (15%)

References

1 Grabherr, M. G. *et al.* Full-length transcriptome assembly from RNA-Seq data without a reference genome. *Nat Biotechnol* **29**, 644-652, doi:10.1038/nbt.1883 (2011).

Supplementary Figures

Figure S1

Distribution of CDR3 length obtained from publically available datasets utilising TraCeR.

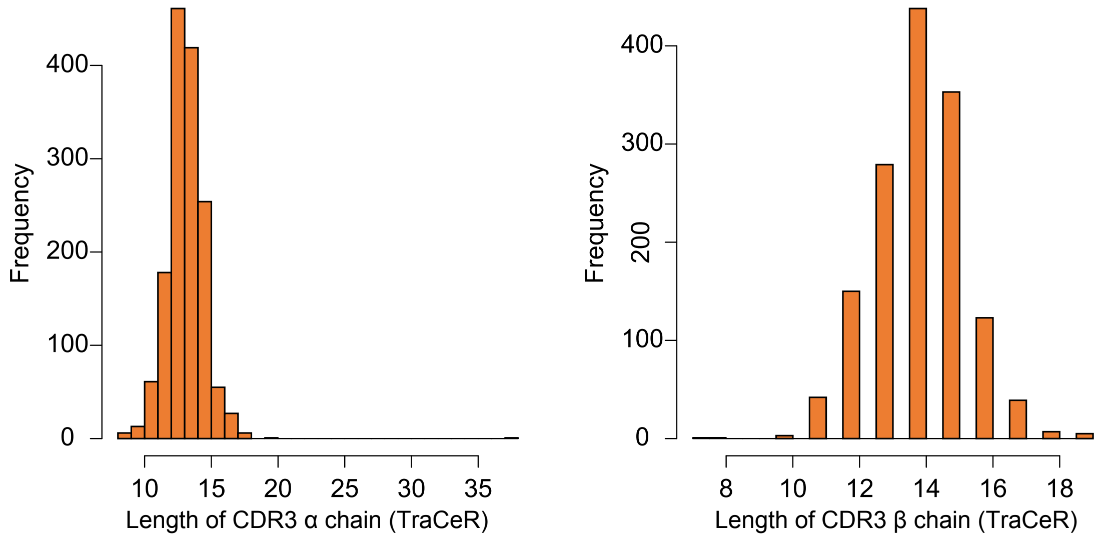

Figure S2

Comparison of the number of genes detected in cells with a single and with two or more αβ pairs, respectively.

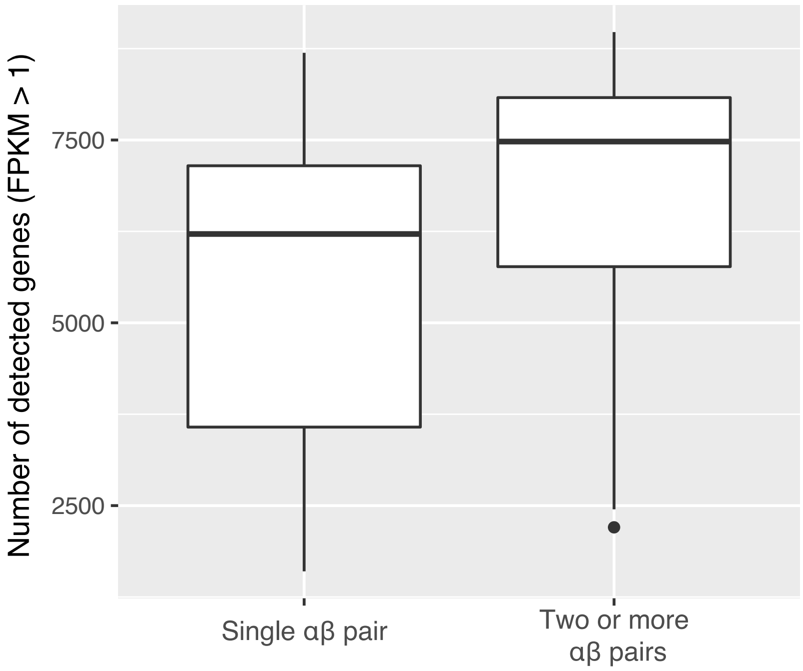

Figure S3

Sigmoidal fit of the TCRαβ reconstruction rate as function of read length and sequencing depth. Simulated data are shown as orange dots.

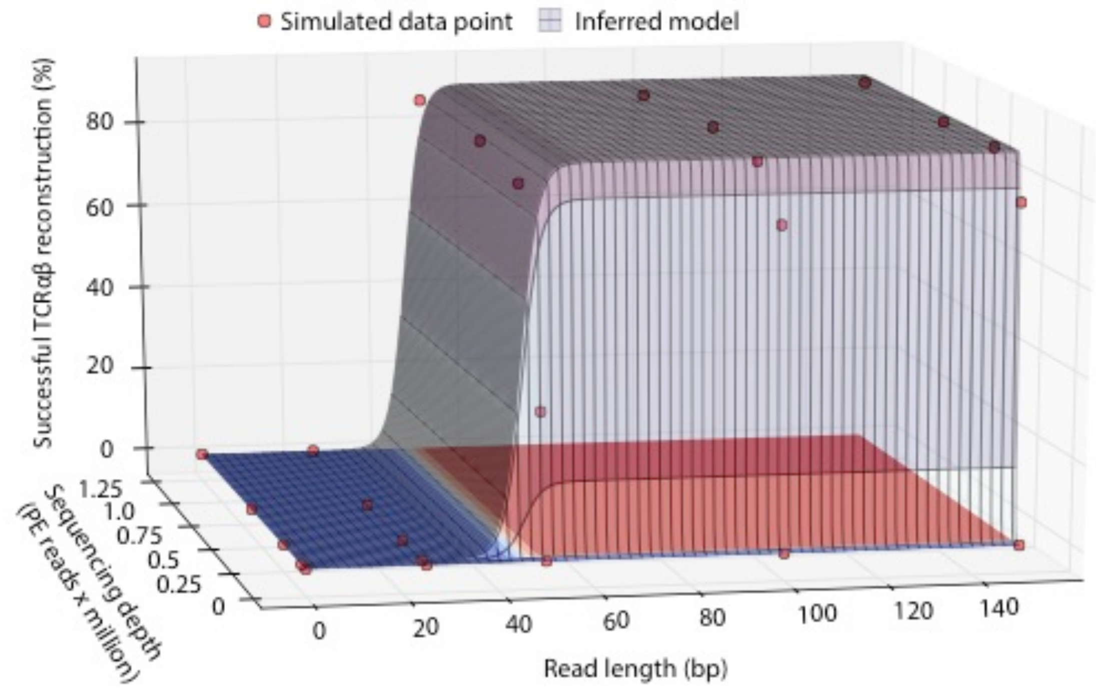

Figure S4

Success rate in detecting double α TCR sequences from simulated scRNA-seq datasets.

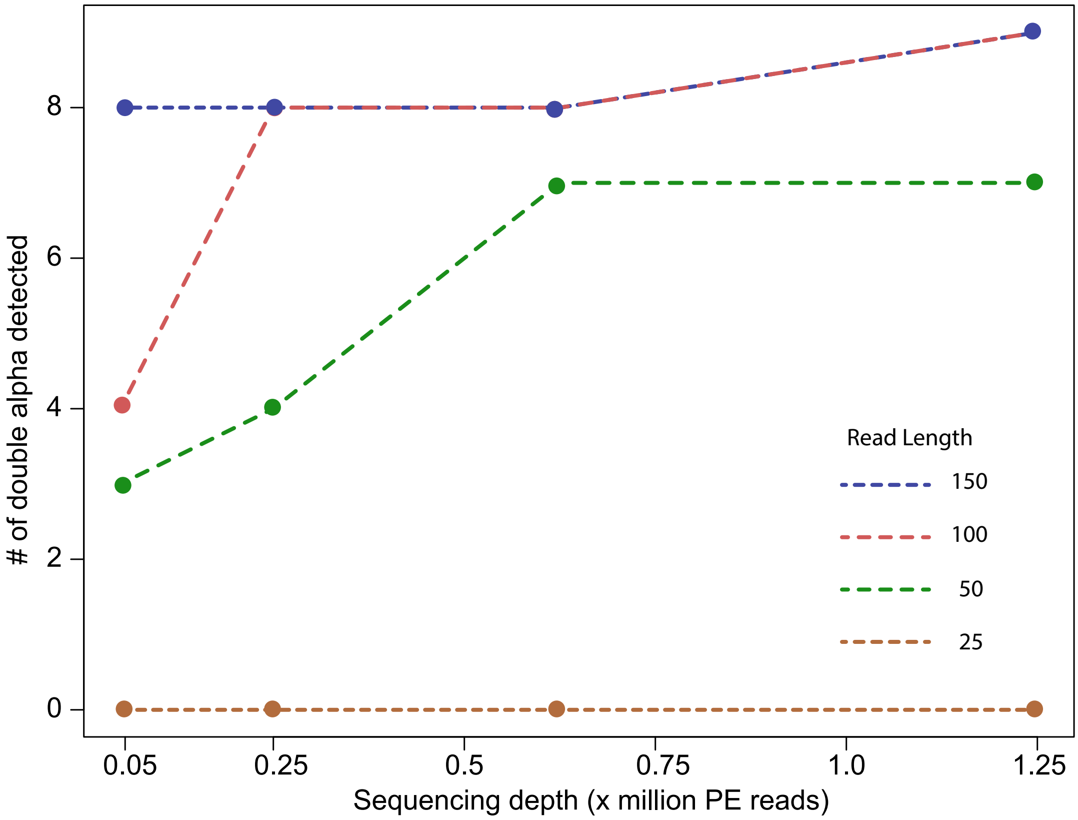

Figure S5

Clustering analysis for the three populations of HCV specific CD8+ T cells in (datasets 1 and 2 in Table 1). **Panels A and B**: Principal Coordinate Analysis of the three subsets of cells by varying read length (50 and 100 bp). Coverage for each dataset was set to 1.25 millions of PE reads per cell. The point colours correspond to the 'ground truth' cell type labels (see legend), and the three point styles correspond to the three identified clusters (circle, triangle and cross). Clustering analysis was performed using CIDR, and forcing the number of clusters to be n=3. **Panels C and D**: the variability within the same cell type (within-class sum of squares) as a function of read length and sequencing depth, for the two in vitro subpopulations, respectively. **Panel E**: Confusion matrices showing the concordance between the CIDR clustering and the ground truth in the 1.25 million read data set, based on 4 different read lengths (25, 50, 100 and 150bp)'.

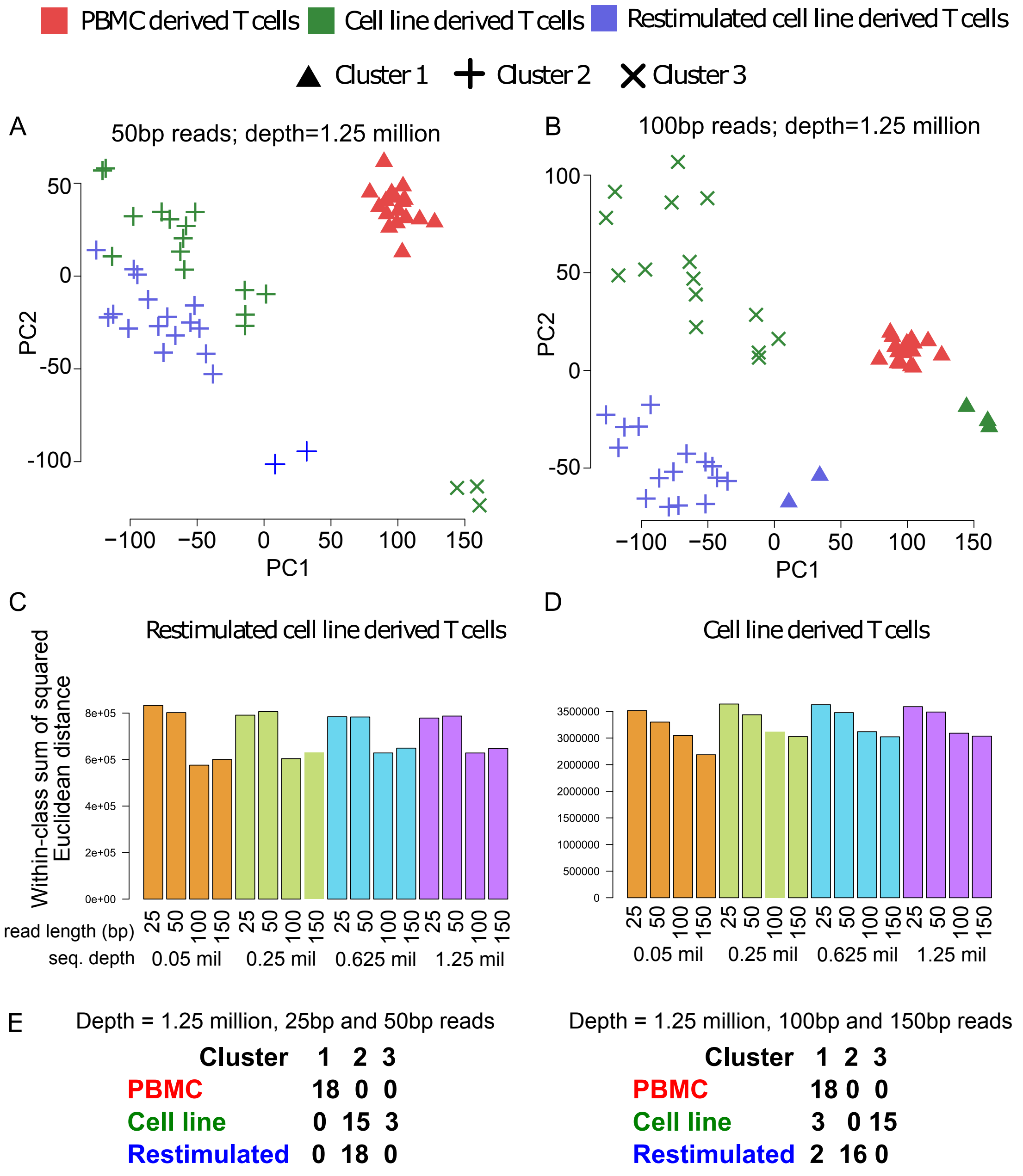

Figure S6

Effect of read length and sequencing depth on gene expression profiles for distinct gene categories using simulated datasets: genes with an average expression level >=100 FPKM, genes with an average expression level <100 FPKM, house keeping genes, and transcription factors.

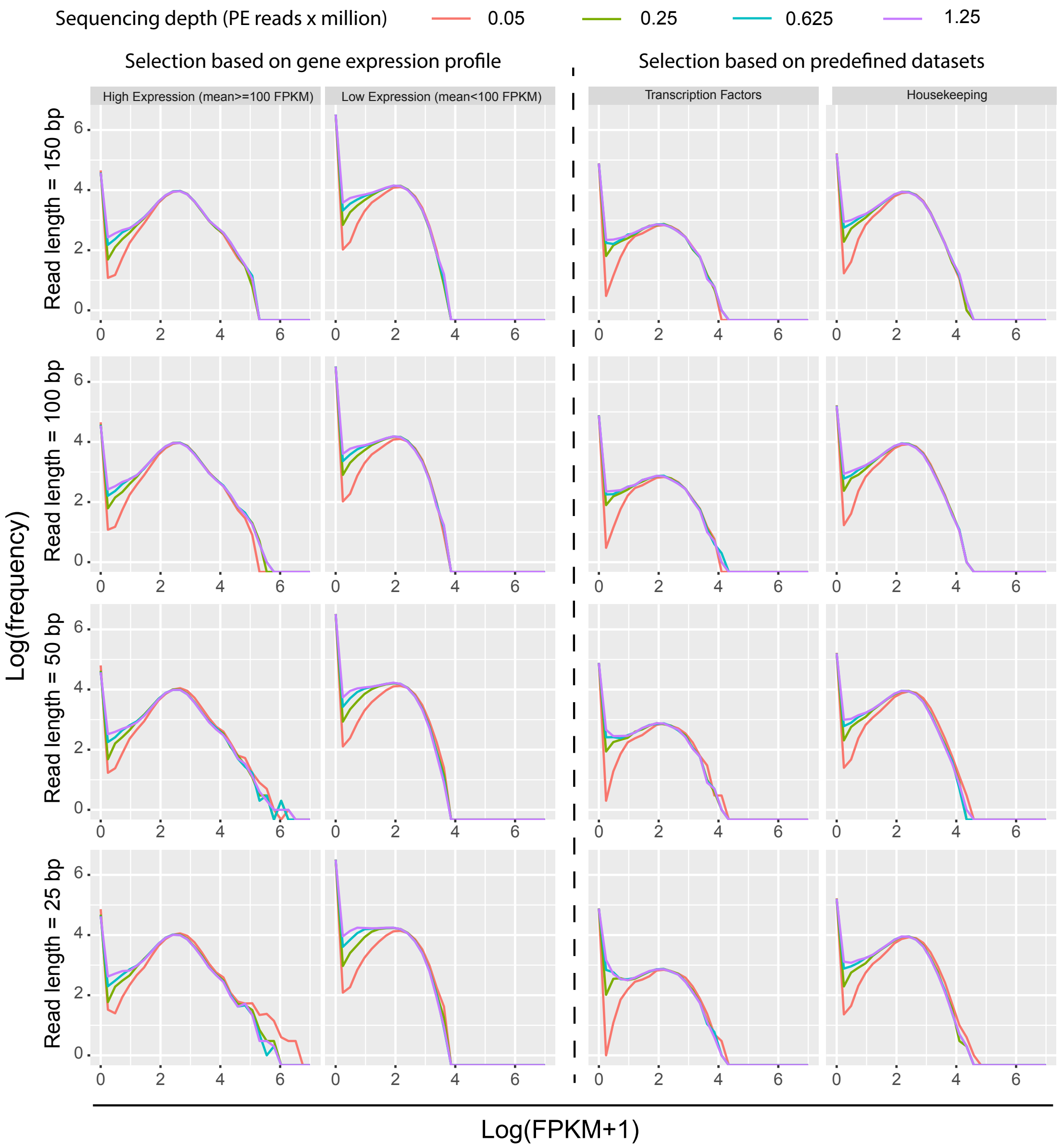

Supplement: Supplementary file 1 — Supplementary Materials [file 41598_2017_12989_MOESM1_ESM.pdf]
